# Fluorescence-Detected Pump–Probe Spectroscopy for Artifact-Free Detection of Stokes Shift Dynamics

**DOI:** 10.1021/acs.jpclett.5c00646

**Published:** 2025-05-09

**Authors:** Hongxing Hao, Pavel Malý, Yang Cui, Maximilian Binzer, Erling Thyrhaug, Jürgen Hauer

**Affiliations:** † Professorship of Dynamic Spectroscopy, Department of Chemistry, TUM School of Natural Sciences, Technical University of Munich, Lichtenbergstraße 4, 85748 Garching, Germany; ‡ Faculty of Mathematics and Physics, Institute of Physics, 138735 Charles University, Ke Karlovu 5, 121 16 Praha 2, Czech Republic

## Abstract

Fluorescence-detected pump–probe (F-PP) spectroscopy is a recently developed method to study excited-state dynamics. F-PP combines the temporal resolution of conventional transient absorption (TA) spectroscopy with the sensitivity of fluorescence detection. In this work, we demonstrate inherently phase-stable F-PP spectroscopy using 20 fs pulses to monitor the ultrafast Stokes shift dynamics of a solvated fluorophore (Y12). We observed a shift in the stimulated emission maximum with a time constant of 84 fs. In contrast to TA, F-PP provides a coherent artifact-free view of this process. Using quantitative signal background subtraction, as discussed in this work, F-PP uncovers the pure stimulated emission spectrum and its ultrafast dynamics. This signal isolation is a clear advantage over TA, where different contributions often overlap heavily. We compare results from F-PP and TA on an equal footing using the same excitation pulses, emphasizing the features and advantages of the F-PP technique.

## Graphical abstract

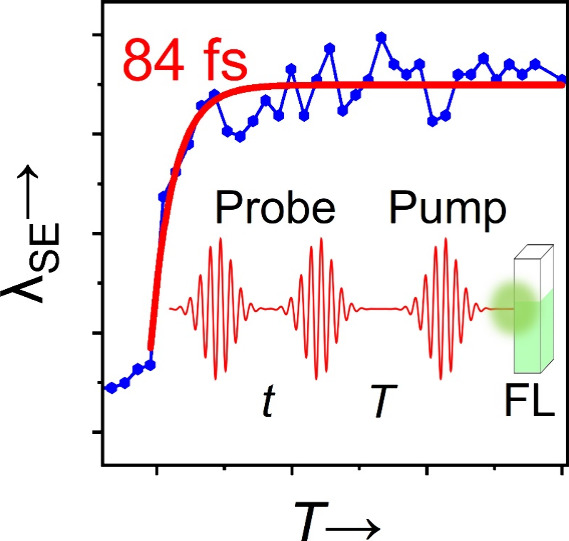

Transient absorption (TA) spectroscopy is a powerful and flexible tool widely used to follow fast photoinduced dynamics. The basic principle of the technique is to monitor the time-dependent changes in ultraviolet/visible (UV/vis) absorption spectra after photoexcitation of a sample with a “pump” pulse. Correlating these spectral changes with molecular dynamics can provide insights into energy relaxation pathways of molecules, aggregates, and solid-state materials.^1–4^ More recently, measuring TA-like spectra via the detection of incoherent fluorescence (FL),^5,6^ a concept inspired by fluorescence-detected two-dimensional electronic spectroscopy (F-2DES), has gained increasing attention.^7–11^ Several limitations in TA can be overcome by FL detection. For example, FL detection is easier to implement in a confocal microscope and is highly beneficial for scattering samples. As a critical advantage for early dynamics, the non-resonant coherent artifacts that often dominate early delay times in TA do not appear in the case of FL detection.^5,12^

The basic principles of action-detected femtosecond spectroscopies have been discussed in detail elsewhere^5,9,13^ and are also of significance in the field of attosecond optics.^14,15^ In brief, F-PP relies on the detection of a sample’s (typically time-integrated) FL signal, recorded as a function of delays between femtosecond pump and probe pulses. The signal of interest can be isolated by, e.g., subtraction of the signals with only pump or probe pulses present, in analogy to subtraction of pump-on/pump-off spectra in conventional TA experiments. While a TA signal is routinely recorded in a spectral dispersion, the resolution of the probe spectrum in F-PP requires interferometry. We achieve this by separating the probe pulse into a phase-locked pair of pulses at a controlled interpulse time separation *t*. A Fourier transformation *t* → ω_*t*_ results in a frequency-resolved transient FL excitation spectrum FL_PP_(ω_*t*_,*T*) at a given pump–probe delay *T*.^5,16,17^ It is possible to further separate the pump pulse into a phase-locked pair with interpulse delay τ, the Fourier transformation of which results in the fluorescence-detected 2D spectrum FL_2D_(ω_τ_,ω_*t*_,*T*), as first demonstrated by Warren and co-workers^18^ and later others.^7,10,19^ Numerous systems have been investigated using F-2DES and F-PP, including molecular dimers,^9^ DNA,^20^ quantum dots,^5^ and light-harvesting complexes.^7,21,22^ One potential limitation of these techniques is that they often involve the incoherent mixing of linear signals within molecular assemblies, which can obscure the extraction of the desired nonlinear response.^23^ In perovskite samples, for instance, incoherent mixing has been shown to contribute to the action-detected 2D signals.^24^ Efforts are currently underway to develop methods to effectively isolate the nonlinear contribution.^25–27^ The implementation of such experiments is typically achieved using either pulse shapers or actively phase-stabilized interferometers.^5,21,28,29^ As spectrally resolved data can be retrieved only via Fourier transformation of an interferogram, minor delay fluctuations in the interferometer may result in large inaccuracies and loss of signal amplitude. We address this problem by using a TWINS common-path interferometer.^30,31^ This interferometer utilizes birefringent crystals, enabling high throughput from the UV to near-infrared (NIR) to generate inherently phase-locked pulse pairs with variable time delay. Its versatility has diverse applications in both linear and nonlinear spectroscopy experiments.^16,32–38^

Despite the similarities outlined above, F-PP and TA have several notable differences. The primary difference is that F-PP results from four perturbative field interactions (fourth-order response), while TA involves only three (third-order response). An immediate consequence is the appearance of additional excited state absorption (ESA)-type signals in F-PP. In the typical case where the fluorescence quantum yield is independent of the excitation wavelength, these additional contributions will result in the exact cancellation of the ESA signals, leaving only stimulated emission (SE) and ground state bleach (GSB) as observable contributions.^9,39^ A second notable difference is apparent for negative pump–probe delays, i.e., where the probe precedes the pump. In TA, the signal will quickly approach zero on a time scale set by the free induction decay as the probe shifts further into negative time. In F-PP, on the other hand, the signal takes the shape of the FL excitation spectrum and approaches zero only slowly, with a loss of the overall signal on a time scale identical to the FL lifetime of the system. Although the measured ultrafast excited-state dynamics remain the same, TA and F-PP spectra will thus appear substantially different, even under identical experimental conditions, as shown below. While evolved data-fitting routines exist to retrieve species-associated spectra,^40^ experimental methods for isolating signal contributions in TA are rare and not without effort.^41^ As discussed quantitatively below, F-PP shows fundamental advantages in this context. For the solvated chromophore studied here, the fluorescence quantum yield is largely independent of the excitation wavelength. Under such circumstances, F-PP suppresses ESA signals and allows the dynamics of the GSB and SE to be fully disentangled. This opens the path to a background-free study of ultrafast Stokes shift dynamics.

The Stokes shift describes the energy difference between a molecule’s absorption and emission peaks and is a key quantity to describe intramolecular energy relaxation and solvent–solute interactions.^42–45^ Experimental studies of ultrafast Stokes shift dynamics have predominantly been conducted using ultrafast fluorescence spectroscopy, where time resolution below ∼100 fs is difficult to achieve due to an intrinsic connection between time resolution and signal strength.^46,47^ The time resolution in a F-PP experiment, on the other hand, is set by the excitation pulse duration, and in analogy to TA, there is no connection to the signal strength. In combination with the ability to isolate SE signals, F-PP is an ideally suited method to study ultrafast reorganization processes in the electronic excited state.

In the F-PP experiment sketched in Figure 1, we employ a Yb:KGW solid-state laser (PHAROS PH2, Light Conversion) to provide a 200 kHz train of 230 fs pulses centered at 1024 nm. After reduction of the repetition rate with an integrated pulse picker, the pulse train is directed to a non-collinear optical parametric amplifier (NOPA Rainbow).^48^ The corresponding TA setup using the same light source is depicted in Figure S2. The resulting broadband pulses are spectrally tunable throughout the visible-to-NIR range (390–950 nm), with spectral bandwidths typically corresponding to Fourier-limited pulses in the 10–20 fs range. For the excitation spectra employed in this work, a simple double-pass prism compressor is sufficient to routinely provide sub-20 fs pulses at the sample position.

**Figure 1:**
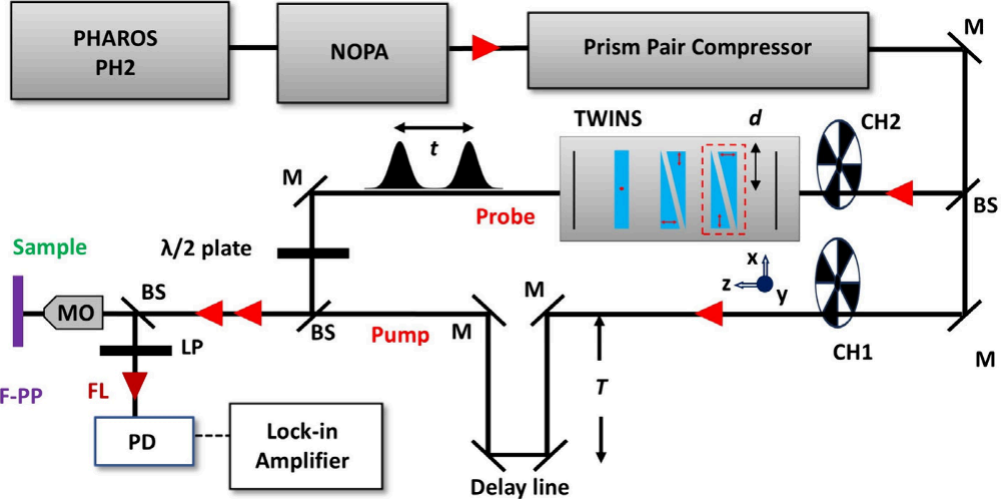
Experimental setup of interferometric F-PP spectroscopy. M, mirror; BS, beam splitter; CH, chopper; MO, microscope objective; LP, long-pass filter; FL, fluorescence; and PD, photodetector. The waiting time delay *T* is controlled by the delay line, while the coherence time *t* is scanned by the TWINS interferometer.

Within the F-PP experiment, we split the beam into separate pump and probe paths. The pump–probe delay *T* is controlled by a linear motor stage (XML350-S, Newport) in the pump arm. The probe beam is directed to a TWINS interferometer (GEMINI-2D, NIREOS) in order to produce the phase-locked pulse pairs necessary for spectrally resolving the probe. The operating principles of this TWINS interferometer have been reported in detail elsewhere.^31^ We combine pump and probe beams on a beam splitter (beamsplitter 103250, Layertec) before the sample and focus the collinear sequence of one pump and two probe pulses using a microscope objective (CFI S Plan Fluor ELWD 20XC, NA 0.45, Nikon). The sample’s FL is collected in back-reflection geometry and routed to a silicon photodetector (DET36A2, Thorlabs). We used a long-pass filter (FELH0850, Thorlabs) to remove the reflected excitation beam. The F-PP measurements were performed at a magic angle orientation, set by a half-wave (λ/2) plate placed in the probe arm.

In general, three signals contribute in a F-PP experiment: the linear FL originating from the sample’s interactions with either the pump or probe pulses alone and the nonlinear FL signal conditional on the sample interaction with both pump and probe pulses. Several schemes have been implemented to isolate this signal (e.g., phase modulation and pump on/off).^5,10^ Here, we rely on amplitude modulation by optical choppers (MC2000B with chopper blades MC1F30, Thorlabs) and a lock-in amplifier (MFIA, Zurich Instruments) to isolate the background-free nonlinear FL signal. The chopper does not introduce chirp into the optical path, facilitating pulse compression without non-standard optics, such as chirped mirrors or grism compressors. The signal from the probe arm alone, chopped with 50% duty cycle, can be decomposed into a cosine series

1 $$I_{\mathrm{pr}}=I_{\mathrm{pr}}\left\{\frac{1}{2}+\frac{2}{\pi }\cos\left(\Omega_{\mathrm{pr}}t\right)+...\right\}$$

where Ω_pr_ is the modulation frequency of the probe beam set by chopper 2 (CH 2 in Figure 1). Similarly, the pump arm was modulated by CH 1 at Ω_pu_, resulting in

$$I_{\mathrm{pu}}=I_{\mathrm{pu}}\left\{\frac{1}{2}+\frac{2}{p}\cos\left(\Omega_{\mathrm{pu}}t\right)+...\right\}$$ 2

The lock-in amplifier demodulates the FL signal at specific frequency components. Given the modulation, the terms at Ω_pu_ and Ω_pr_ are dominated by the linear fluorescence FL_lin_^(2)^(ω_*t*_) response to the pump and probe pulses, respectively.

3 $${{\mathrm{FL}}_{\mathrm{lin}}}^{\left(2\right)}\left(\omega_{t}\right)\propto {{\mathrm{FL}}_{\mathrm{pu}}}^{\left(2\right)}I_{\mathrm{pu}}\cos\left(\Omega_{\mathrm{pu}}t\right)+{{\mathrm{FL}}_{\mathrm{pr}}}^{\left(2\right)}\left(\omega_{t}\right)I_{\mathrm{pr}}\left(\omega_{t}\right)\cos\left(\Omega_{\mathrm{pu}}t\right)$$

The desired nonlinear fluorescence signal, FL_PP_^(4)^(ω_*t*_,*T*), arises from the sample interacting with both the pump and probe pulses and is proportional to

4 $$\begin{array}{l} {{\mathrm{FL}}_{\mathrm{PP}}}^{\left(4\right)}\left(\omega_{t},T\right)\propto {\mathrm{FL}}^{\left(4\right)}\left(\omega_{t},T\right)I_{\mathrm{pu}}I_{\mathrm{pr}}\left(\omega_{t}\right)\cos\left(\Omega_{\mathrm{pu}}t\right)\cos\left(\Omega_{\mathrm{pr}}t\right)\\ =\frac{1}{2}{\mathrm{FL}}^{\left(4\right)}\left(\omega_{t},T\right)I_{\mathrm{pu}}I_{\mathrm{pr}}\left(\omega_{t}\right)\left[\cos\left(\Omega_{\mathrm{pu}}+\Omega_{\mathrm{pr}}\right)t+\cos\left(\Omega_{\mathrm{pu}}-\Omega_{\mathrm{pr}}\right)t\right] \end{array}$$

The desired nonlinear FL signal FL_PP_^(4)^(ω_*t*_,*T*) can thus be isolated by the lock-in amplifier at the sum and difference frequency of the individual beam modulations. For clarity, we indicate the order of perturbation as a superscript in parentheses to distinguish between desired fourth-order and undesired linear contributions. Note that the square-wave amplitude modulation of optical choppers results in signals at the fundamental modulation frequency and at all its harmonics. A careful choice of demodulation frequencies is thus required to avoid the pollution of the desired signal by harmonics of the pump and probe frequencies. In the experiments described in the following, the repetition rate of the primary laser source was set by an internal pulse picker to 200 kHz/9 = 22.2 kHz. Both choppers were triggered by the TTL signal from the laser source. The modulation frequency of the pump was set at 1.59 kHz (subharmonic of 14:22.2 kHz/14), while the probe modulation frequency was set to 2.78 kHz (subharmonic of 8:22.2 kHz/8).

We used the dye BTP-4F-12 (Y12) as a test sample. Y12, a commonly used non-fullerene acceptor in organic solar cells,^49^ is a convenient model system due to its photostability, large absorption cross section, and approximately 70% FL quantum yield. We show the molecular structure as well as the optical absorption and emission spectra of Y12 in a chloroform (TCM) solution in Figure 2b. The peak assignments of the absorption spectrum have been discussed elsewhere.^50,51^ The FL spectrum exhibits a pronounced Stokes shift of ∼48 nm. We use spectrally degenerate pump and probe pulses with a spectrum shown as a shaded red area in Figure 2b. To compare and contrast the F-PP experiment with a more conventional technique, we perform TA experiments using the same excitation pulse (pulse measurement in Figures S3 and S4) and a white light probe.

**Figure 2:**
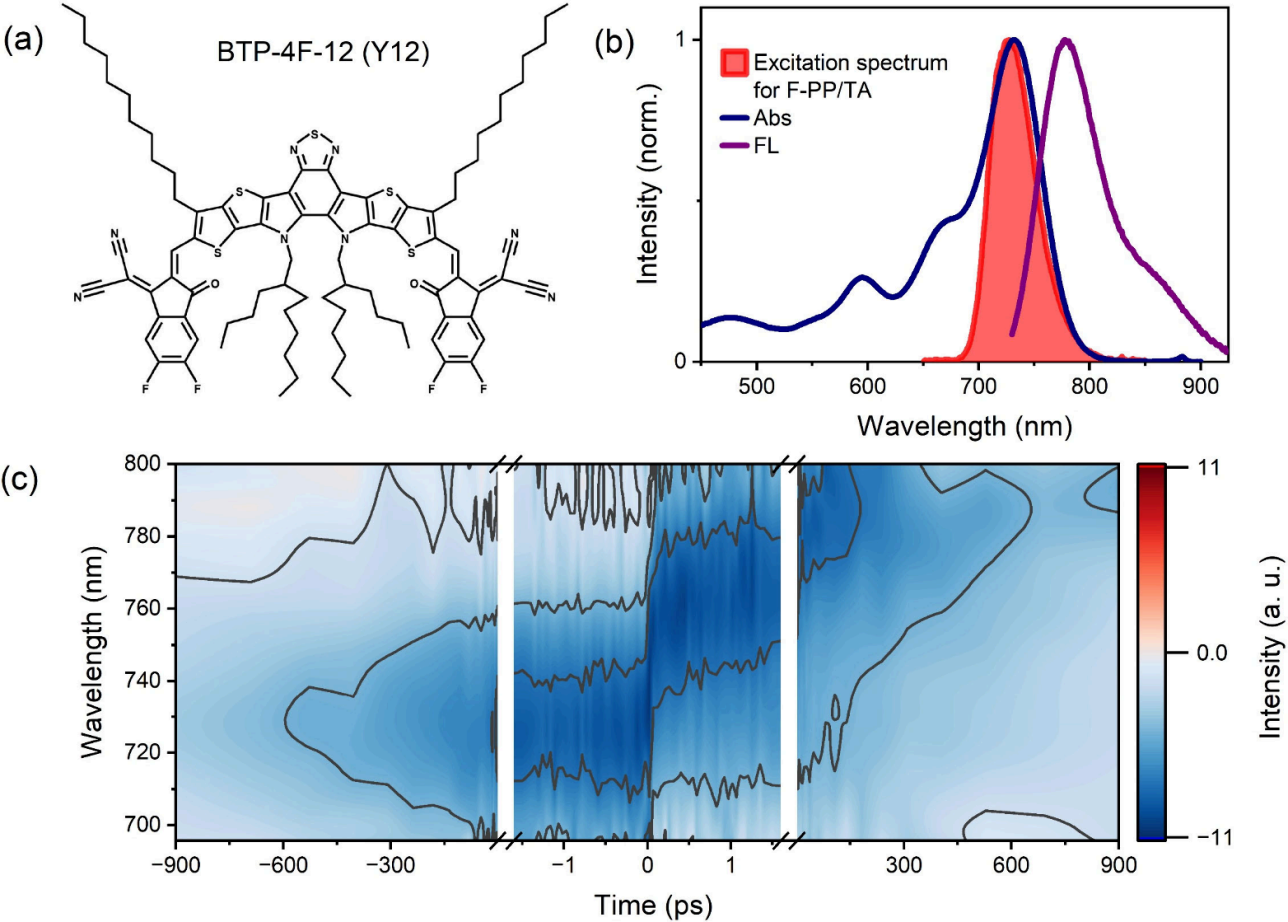
(a) Molecular structure of BTP-4F-12 (Y12). (b) Absorption (blue) and emission (purple) spectra of Y12 in TCM. The red color shows the pump/probe pulse spectrum in F-PP and the pump pulse in TA experiments. (c) F-PP spectra of Y12 in TCM.

We show the 2D detection wavelength versus pump–probe delay F-PP maps in Figure 2c. As expected given previously reported results,^5^ and unlike conventional TA, we observe a strong GSB-like signal before time zero. In the limit of broadband excitation,^5^ these negative-time dynamics report essentially on the ground-state recovery only, with no contributions from excited-state dynamics (see Figure S1 for details). At positive pump–probe delays, on the other hand, the F-PP spectrum contains both GSB and SE contributions and, thus, reports directly on the dynamics of the excited state. Here, by tailoring the excitation spectrum to effectively probe the SE region, we observe a strong red shift of the signal caused by the Stokes shift.

As the spectra at *T* < 0 reflect the GSB line shape or more accurately the FL excitation spectrum, we can further isolate the SE contribution for a spectrum at *T* by subtracting an appropriately weighted spectrum at each −*T*.^5,27^ In general, the desired scaling factor for the spectrum at −*T* is derived from analysis of the excitation pathways, as shown in Figure S1. When the F-PP signal is divided by probe’s spectrum *I*(ω_*t*_) (see eq 4), the signal at positive times can be expressed as (see the *T* > 0 part of Figure S1)­

$$\mathrm{F‐PP}\left(\omega_{t},T>0\right)=\mu_{1}^{2}\left(\underset{O_{\mathrm{abs}}}{\underset{︸}{\int d\omega_{\tau }\alpha_{1}\left(\omega_{\tau }\right)\cdot I\left(\omega_{\tau }\right)}}\right)\left\{\mu_{1}^{2}\alpha_{1}\left(\omega_{t}\right)+\mu_{1R}^{2}\sigma_{1R}\left(\omega_{t},T\right)\right\}$$ 5

where α_1_(ω_*t*_) is the absorption line shape, σ_1*R*_(ω_*t*_,*T*) is the SE line shape with time-dependent Stokes shift in *T*, and *I*(ω_*τ*_) is the laser spectrum. μ_1_^2^ and μ_1*R*_^2^ are the GSB and SE transition strength, respectively. At negative times, the signal is given by (see the *T* < 0 part of Figure S1)­

6 $$\mathrm{F‐PP}\left(\omega_{t},T<0\right)=\mu_{1}^{2}\left\{\mu_{1}^{2}O_{\mathrm{abs}}+\mu_{1R}^{2}\underset{O_{\mathrm{SE}}\left(T\right)}{\underset{︸}{\left(\int d\omega_{\tau }\sigma_{1R}\left(\omega_{\tau },T\right)\cdot I\left(\omega_{\tau }\right)\right)}}\right\}\alpha_{1}\left(\omega_{t}\right)$$

At *T* > 0, the signal is thus weighted by the overlap of the laser excitation and absorption spectra $O_{\mathrm{abs}}=\int d\omega_{\tau }\alpha_{1}\left(\omega_{\tau }\right)\cdot I\left(\omega_{\tau }\right)$ . At *T* < 0, the probe excites first and the signal scales with both *O*_abs_ and the overlap of the excitation and (time-dependent) SE spectra, $O_{\mathrm{SE}}\left(T\right)=\int d\omega_{\tau }\sigma_{1R}\left(\omega_{\tau },T\right)\cdot I\left(\omega_{\tau }\right)$ . Based on these definitions, we can now derive an expression for the pure SE spectrum. If we assume that the dipole moments for absorption and SE are equivalent (i.e., μ_1_^2^ = μ_1*R*_^2^), we obtain the following expression for the difference between a F-PP spectrum at *T* and −*T*:

$$\mu_{1}^{4}O_{\mathrm{abs}}\left\{\sigma_{1R}\left(\omega_{t},\;T\right)+\alpha_{1}\left(\omega_{t}\right)[1-f\frac{O_{abs}+O_{SE}(T)}{O_{abs}}]\right\}\;$$ 7

where *f* is the scaling factor for which we want to find an expression. We know the strength and shape of the GSB signal, α_1_(ω_*t*_), as it is given by the F-PP signal at negative *T* values. If we want to remove the GSB contributions from the F-PP signal at positive times, then the scaling factor *f* must be set correctly. According to eq 7

8 $$f=O_{\mathrm{abs}}/\left(O_{\mathrm{abs}}+O_{\mathrm{SE}}\left(T\right)\right)$$

cancels out the prefactor of α_1_(ω_*t*_) and allows for the desired removal of GSB contributions from F-PP­(ω_*t*_,*T* > 0). Equation 8 gives us the definition of *f*, but it does not directly lead to an experimental procedure to determine it. This becomes clear when considering that *O*_abs_ is readily determined but *O*_SE_(T) is not, especially in its *T* dependence. We now discuss limiting cases for *f* in which *O*_SE_(*T*) is known. In the hypothetical case of impulsive excitation and probe pulses, all diagrams in Figure S1 contribute equally and *O*_abs_ ≈ *O*_SE_(*T*), leading to *f* = 0.5, as used in a previous work.^5^ For the finite-bandwidth spectrum, the equality between *O*_abs_ and *O*_SE_(*T*) does not hold generally. We can, however, still determine *f* for limiting cases of *T*. For values of *T* prior to reorganization dynamics, the SE spectrum can be approximated by a (mirrored) absorption spectrum. As a result, *O*_abs_ ≈ *O*_SE_(*T*) holds again, leading to *f* = 0.5. For later values of *T* after the Stokes shift has occurred, the SE spectrum will coincide with the fluorescence spectrum. For the case of Y12 in the TCM studied here, this leads to *f* = 0.76. We have thus established two limiting cases for *f* for early and late values of *T*: 0.5 < *f* < 0.76. We can readily validate these limits by evaluating *f* for a spectral range free of SE and, thus, solely defined by GSB. In such a case, σ_1*R*_(ω_*t*_,*T*) must be zero, which leads to the following expression for the ratio of F-PP signals at *T* < 0 and *T* > 0:

9 $$\frac{\mathrm{F‐PP}\left(\omega |\sigma_{1R}\left(\omega \right)=0,T<0\right)}{\mathrm{F‐PP}\left(\omega |\sigma_{1R}\left(\omega \right)=0,T>0\right)}=\left(\frac{O_{\mathrm{abs}}+O_{\mathrm{SE}}\left(T\right)}{O_{\mathrm{abs}}}\right)=f^{-1}$$

The factor *f* determined in this way for Y12 in TCM amounts to *f* = 0.65 on average, which is well within the limits for *f* discussed above, see also Figure S5. Now that we have established the validity of 0.5 < *f* < 0.76, the question arises if the Stokes shift dynamics is affected by the choice of *f*. Figure 3a shows the corrected F-PP spectra for *f* = 0.65. When interested in the Stokes shift dynamics or the *T* dependence of σ_1*R*_(ω_*t*_,*T*), we extract it by plotting the peak maximum as a function of *T* for *T* > 0. We show the extracted Stokes shift dynamics using the intermediate value (*f* = 0.65) in Figure 3b. This leads to an exponential rise time of 84 fs; see the red line in Figure 3b. The results of the fit are basically identical when using *f* = 0.5 and 0.76, as shown in Figure S6. This underlines the weak dependence on *f* and the general robustness of the approach. We note that the subtraction of F-PP­(*T* > 0) and F-PP­(*T* < 0) is broadly applicable for highlighting excited-state dynamics, in our case the dynamic Stokes shift.^27^ This method can be readily extended to action-detected techniques, such as photocurrent and photoelectron detection schemes.^52–54^ Importantly, contributions due to pulse overlap effects are minimal in F-PP (see Figures 2c and 3a around time zero), which aids the analysis of ultrafast phenomena. The straightforward procedure to analyze the ultrafast Stokes shift dynamics that we describe here makes F-PP a valuable tool in this context. In comparison to the more widespread fluorescence upconversion,^55^ the spectral coverage is given by the probe pulse, which can be replaced by a white light continuum^38^ and not by the properties of the upconversion crystal.

**Figure 3:**
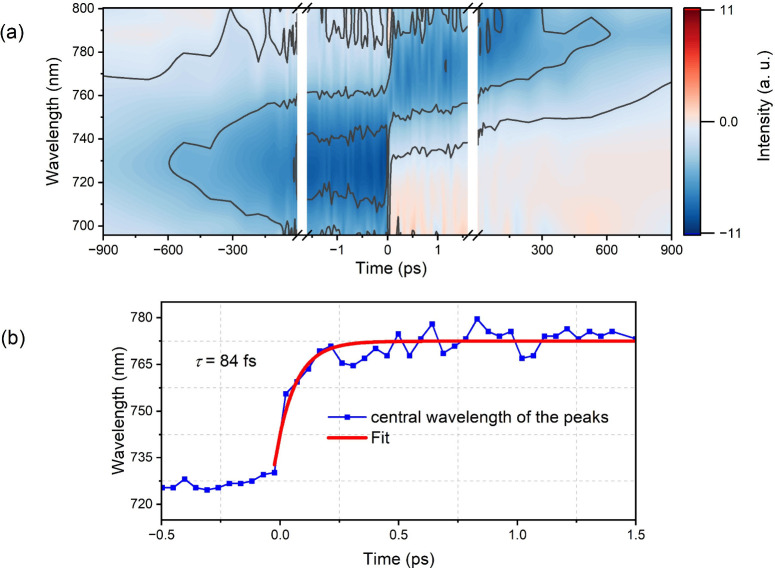
(a) To quantify the Stokes shift dynamics, we first subtract the GSB component (see the main text for details, using *f* = 0.65), measured at *T* < 0 for every *T* > 0. Second, we extract the wavelength of the peak maximum and plot it in panel b. A 84 fs exponential rise accurately describes the early dynamics.

In Figure 4, we compare results from the TA and F-PP of Y12 using identical excitation pulses. TA maps and a sketch of the experiment can be found in Figures S7 and S2, respectively. The TA signal at *T* = 4 ps is depicted as a gray line and shows a negative peak at 750 nm and a positive peak at 875 nm. The latter is readily attributed to an ESA transition, while the former (negative) feature originates from GSB and SE, as seen by comparison to the inverted absorption and emission spectra (light-shaded violet and olive curves in Figure 4).^51,56^ Clearly, these features are difficult to separate due to strong spectral overlap.

**Figure 4:**
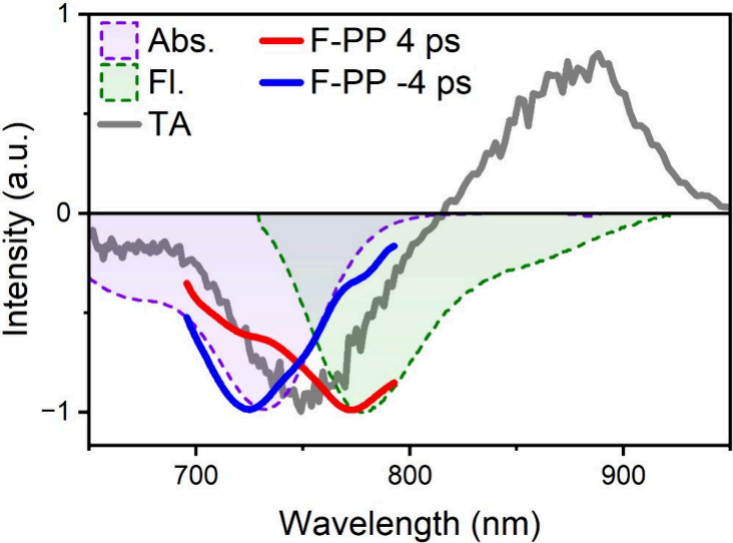
TA and F-PP spectra slices at 4 ps and negative time (−4 ps) of F-PP. Shaded areas are absorption (violet) and FL (olive) spectra of Y12 in TCM with inverted signs.

The F-PP spectrum for negative times (blue line in Figure 4) shows only GSB-like contributions, as witnessed by the good agreement between the F-PP signal at *T* = −4 ps and the absorption spectrum (violet) in Figure 4. According to expectation (see Feynman-diagrams in Figure S1), the F-PP signal at *T* = 4 ps shows a peak and a shoulder, corresponding to the SE/FL maximum at 773 nm and the GSB maximum at 725 nm, respectively. At 4 ps, most of the spectral relaxation has already occurred, explaining the good overlap between the F-PP curve and the FL spectrum (olive filled curve). An obvious mismatch exists between the independently measured TA spectrum (gray curve) and the F-PP at 4 ps (see gray vs red curve in Figure 4 and Figure S8). The reason is that F-PP is free of ESA contributions,^5^ while the TA spectrum is defined by the overlap of GSB and SE (both negative) and ESA (positive). This, in turn, means that F-PP can help disentangle TA signals and retrieve pure ESA spectra. It was shown recently that polarization control in TA may also alleviate these spectral congestion issues.^41^

The advantage of ESA-free spectra in F-PP also becomes obvious when comparing results from global analysis on TA and F-PP; both data sets were fitted using an open-source global analysis package.^57^ The combined amplitude information gives rise to decay-associated spectra (DAS), offering insight into individual component growth and decay behaviors. We present the DAS in panels a and b of Figure 5, while evolution-associated spectra (EAS) are displayed in Figure S9. The fit of the F-PP data reveals two distinct lifetime components of τ_1_ = 2.97 ps and τ_2_ = 1.08 ns, where the long-lived component’s lifetime was fixed to the fluorescence lifetime of Y12. We note a discrepancy in the relative weight of GSB and SE in the long-time-delay F-PP relative to our expectations given the GSB and steady-state FL. Such discrepancies have been observed earlier in action-detected experiments,^8^ where it was assigned to imperfect cancellation of ESA resulting in line shape distortions. For the simple molecular system investigated here, we have no evidence for the breakdown of Kasha’s rule required for this mechanism. An alternative explanation is that the spectra are influenced by spatial chirp of the NOPA excitation pulses due to technical limitations.^58^ We are currently investigating the influence of the spatial chirp on F-PP signals.

**Figure 5:**
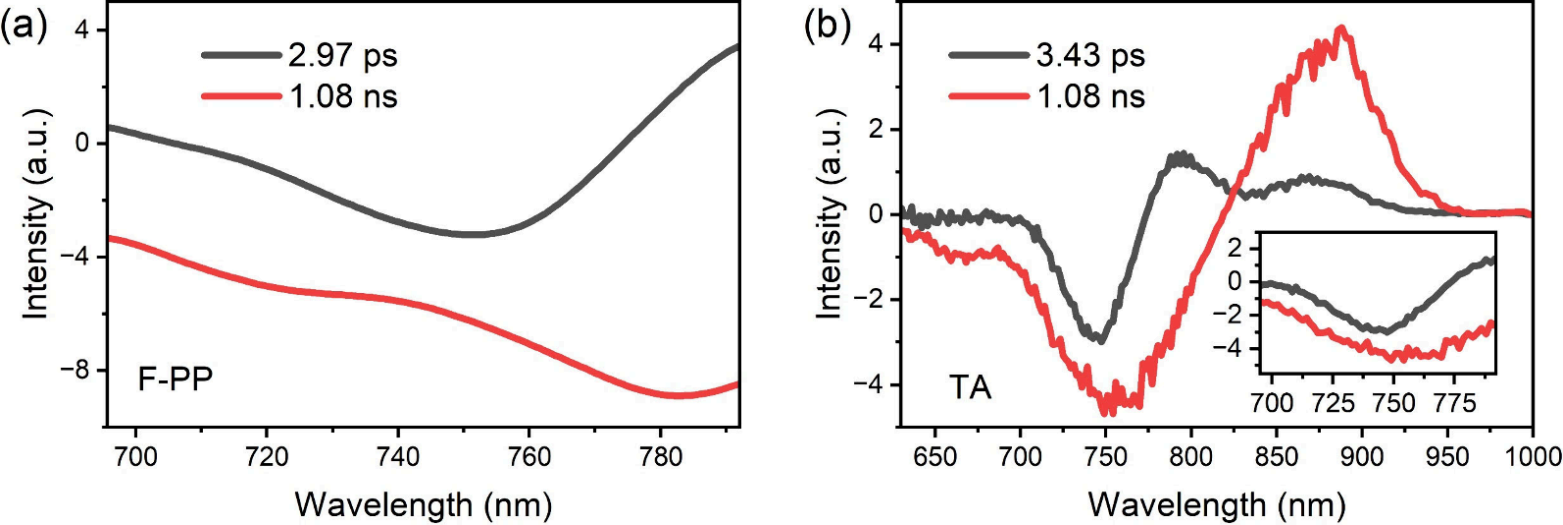
DAS of Y12 in TCM: (a) F-PP and (b) TA DAS.

The fitting results for the TA data reveal a similar lifetime for the initial component with τ_1_ = 3.43 ps. The first DAS component of the F-PP data exhibits a decay centered at around 745 nm, accompanied by a rise at lower energies around 780 nm. This spectral behavior is characteristic of molecular structural relaxation, suggesting a rapid conformational adjustment following photoexcitation. The longer lifetime component, fixed at 1.08 ns, corresponds to the ground-state recovery after structural relaxation, as independently confirmed by time-correlated single-photon counting (TCSPC) measurements, shown in Figure S10. Within the same spectral range, F-PP and TA exhibit similar time constants and number of species but different spectral shapes. While the DAS for F-PP show clear evolution on the red edge of the spectrum, concomitant with Stokes shift, the same signatures are much more subtle in TA (see the inset of Figure 5b). We attribute the increased clarity of the F-PP data to the overlap of ESA and SE (GSB) in TA, while ESA is absent if F-PP.

In summary, we have presented a passively phase-stabilized approach to ultrafast F-PP spectroscopy based on conventional optics. We demonstrate that the chopper-based amplitude modulation method is applicable for F-PP, enabling the efficient and online removal of undesired fluorescent backgrounds. As a proof-of-principle, we measured F-PP spectra of dye Y12 in TCM. Our results suggest that F-PP is a powerful method to study ultrafast excited-state relaxation dynamics. We demonstrate the isolation of SE-type signals in F-PP, which leads to an unperturbed view of ultrafast Stokes shift dynamics. Conventional TA spectroscopy carried out under the same excitation conditions did not recover the same Stokes shift dynamics due to convoluted spectral signatures. The unique capabilities of F-PP described here can complement TA, uncovering otherwise hidden ultrafast reorganization processes.

## Supplementary Material



## Notes

Additional experimental details, double-sided Feynman diagrams in F-PP spectroscopy, TA experimental design, pulse characterization in F-PP and TA experiments, F-PP and TA map, TA time-dependent spectra, evolution-associated spectra of F-PP and TA, and fluorescence lifetime decay of Y12 (PDF)
