# Supplementary material for: Fluorescence-Detected Pump–Probe Spectroscopy for Artifact-Free Detection of Stokes Shift Dynamics
Source: J Phys Chem Lett. 2025 May 9;16(20):4861–8. doi: 10.1021/acs.jpclett.5c00646 (PMC12105010; doi:10.1021/acs.jpclett.5c00646)
Supplement: Supplementary file 1 [file jz5c00646_si_001.pdf]

Supporting Information for

Fluorescence-Detected Pump–Probe Spectroscopy for Artefact-Free Detection of Stokes Shift Dynamics

Hongxing Hao[1], Pavel Malý[2], Yang Cui[1], Maximilian Binzer[1], Erling Thyrhaug[1], Jürgen Hauer[1]**

[1]Technical University of Munich, TUM School of Natural Sciences, Department of Chemistry, Professorship of Dynamic Spectroscopy, Lichtenbergstrasse 4, 85748 Garching, Germany

[2]Faculty of Mathematics and Physics, Institute of Physics, Charles University, Ke Karlovu 5, 121 16 Praha 2, Czech Republic

Corresponding Author

* erling.thyrhaug@tum.de

* juergen.hauer@tum.de

1. Additional experimental details

1.1 Steady-state spectroscopy

The absorption and emission spectra of BTP-4F-12 (Y12) in chloroform (TCM) were performed in a 1 cm fused silica cuvette. Absorption spectra were measured using a PerkinElmer Lambda 365 UV–Vis spectrophotometer, whereas fluorescence spectra were recorded with an Edinburgh FS5 spectrofluorometer. In fluorescence measurements the maximum optical density was 0.07 in order to avoid inner filter effects.

1.2 Collinear frequency-resolved optical gating (cFROG)

We use the compressed output of the NOPA to follow the ultrafast photoinduced dynamics. The compression of pulses was characterized at the sample position of F-PP experiments using collinear frequency-resolved optical gating (cFROG), which allows retrieval of pulse intensity and phase in both frequency and time domain. [1, 2] The cFROG measurements were conducted using a 10 μm thick BBO crystal. The frequency-resolved interferometric autocorrelation trace was measured with a fiber-coupled spectrometer in the forward direction, where the second harmonic generation was detected. In order to compensate for the dispersion of the front cuvette window, a 1.25 mm quartz compensation plate was inserted when measuring the cFROG trace. We show typical pulse-characteristics obtained using a FROG retrieval software (FROG 3.2.2, Femtosoft Technologies) in Fig. S4.

2. Double-sided Feynman diagrams in F-PP spectroscopy

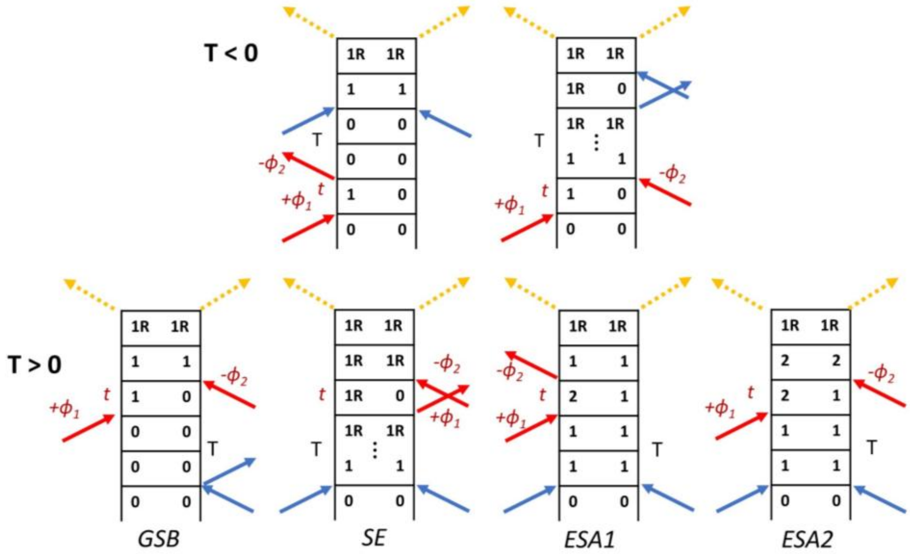

Fig. S1. Double-sided Feynman diagrams for F-PP spectroscopy techniques. The contributions pathways are indicated at T < 0 and T > 0. Pump pulses are depicted in blue, probe pulses in red, and signals in orange. The incoming arrows indicate interaction with the pulses, outgoing arrows indicate signal emission, time flows upwards, and numbers indicate manifolds in the elements of the system density matrix. Initially, the system is assumed in the ground state $|0\rangle$. The pump pulse generates a population of the system states, which then evolves over a waiting time T. Under the influence of the pump and probe pulses, the system can transition to the first excited state $|1\rangle$ or even a higher excited state $|2\rangle$. Specifically, we use $|1R\rangle$ to denote the energy level associated with its fluorescence, where the signal is emitted and detected during the system evolution. For T <0, the probe pulse pair scan results in the fluorescence excitation spectrum, corresponding to the GSB lineshape. When T > 0, the probe can either be absorbed (GSB-type signal), induce stimulated emission (SE), or cause a transition into a higher excited state (ESA-

type). The additional ESA2 pathway is represented because of the additional interaction with the last probe pulse field. We noted that the spectral frequency corresponds to the energy difference between the constituent states, which explains the observed Stokes shift when stimulated emission (SE) occurs.

3. Experimental design in F-PP and TA experiments

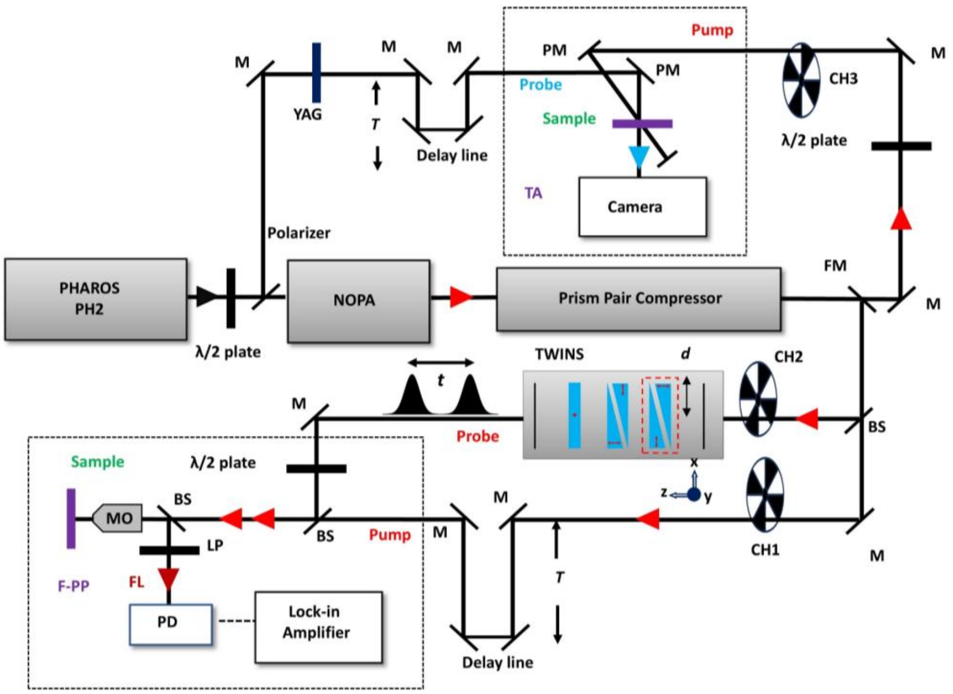

Fig. S2: Experimental setup of the interferometric F-PP spectroscopy and TA spectroscopy. Switching the experiment between TA and F-PP requires a flip mirror. M: Mirror; PM: Parabolic Mirror; CH: Chopper; FM: Flip Mirror; BS: Beam Splitter; MO: Microscope Objective; LP: Longpass Filter; FL: Fluorescence PD: Photodetector; The pump beam is chopped to meet the requirement of heterodyne detection in the TA experiment, isolating the differential absorption

signal. And the probe beam (white light) is delayed by a motor stage (Newport). Both beams are focused to the sample position using parabolic mirrors. The TA signal is detected using a camera.

4. Pump pulse characterization in Transient absorption (TA) experiment

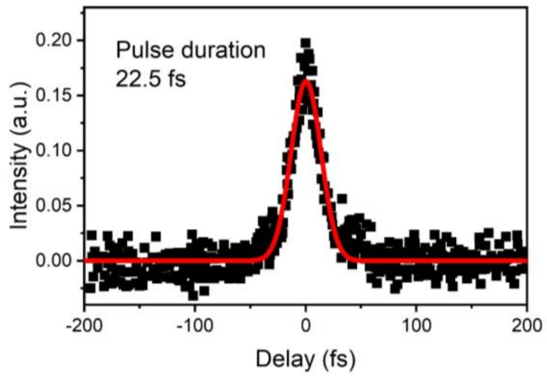

Fig. S3. Pump pulse characterization in the TA experiment by autocorrelator, pulse duration ca. 22.5 fs with Gaussian Fit.

5. Pulse characterization in F-PP experiment

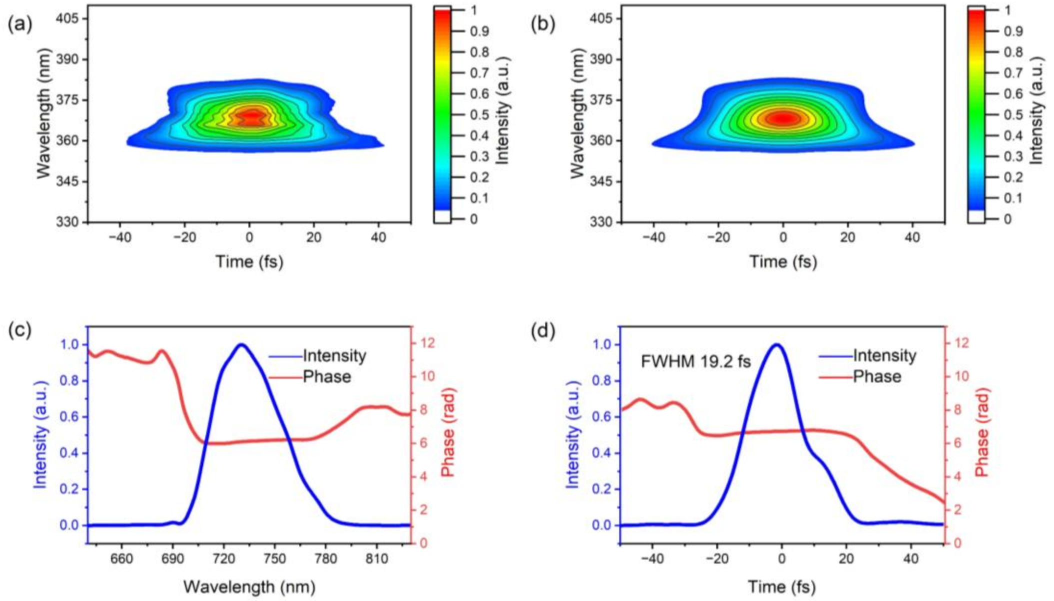

Fig. S4. Characterization of the laser pulse using cFROG. (a) Measured FROG trace. (b) Retrieved FROG trace. (c) The retrieved spectrum and spectral phase. (d) The retrieved pulse shape and temporal phase, pulse duration ca. 19.2 fs.

6. Scaling factor in F-PP experiment

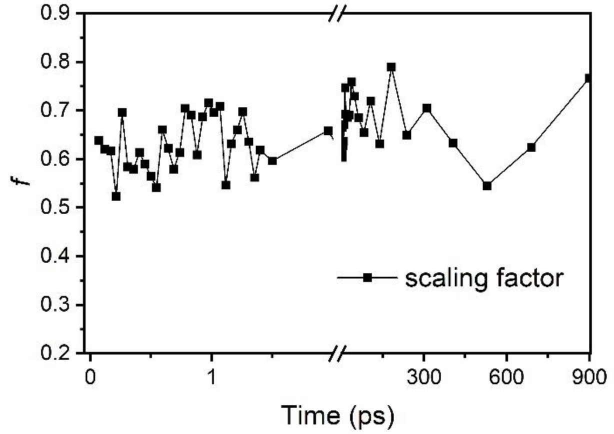

Fig. S5. The value of f at each T. Each f was calculated using Equation (8) in the main text.

7. Characterization of Stokes dynamics

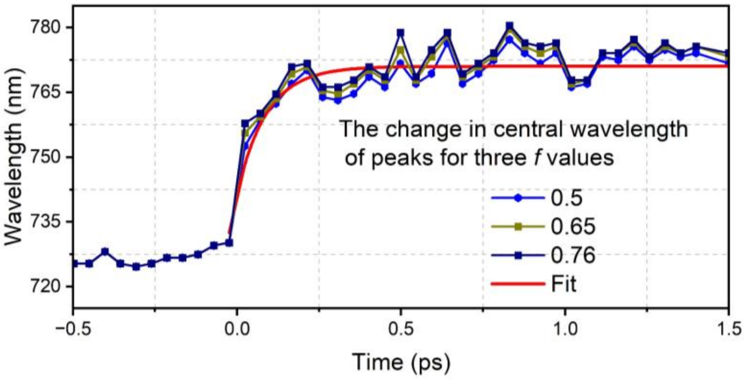

Fig. S6. Characterization of Stokes dynamics using $f = 0.5, 0.65$ and $f = 0.76$, respectively. The fit is shown for $f = 0.65$ and gives an exponential rise time of 84 fs. For $f = 0.5, 0.76$ we obtain 90 fs and 78 fs, respectively.

8. Comparison between TA and FPP

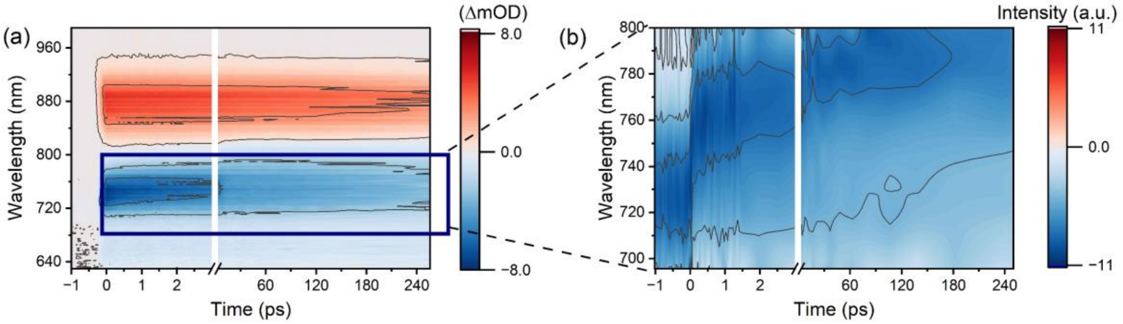

Fig. S7: (a) TA map of Y12 in TCM and (b) F-PP map at positive time.

9. TA time-dependent spectra

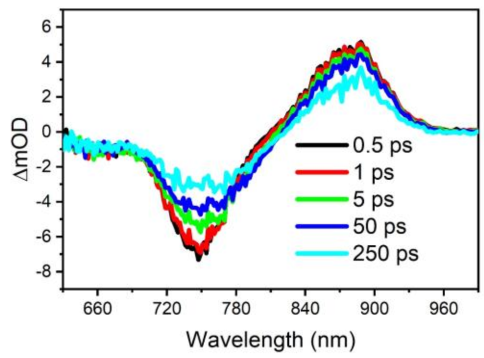

Fig. S8. TA spectra of the Y12 in TCM at different delay times.

10. Evolution-associated spectra

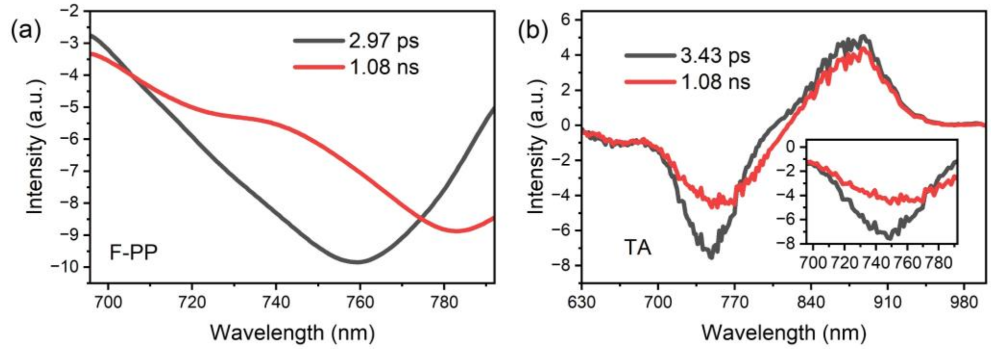

Fig. S9. Evolution-associated spectra (EAS) of the Y12 in TCM solution. (a) F-PP and (b) TA EAS.

11. Fluorescence lifetime decay

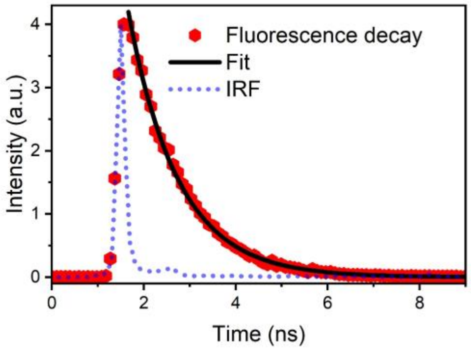

Fig. S10. Fluorescence decay of the Y12 in TCM, excited at 450 nm, and the instruments response function (IRF) curves. A deconvoluted single exponential-decay fitting curve is also shown with a 1.08 ns lifetime.

REFERENCES

(1) Amat-Roldán, I.; Cormack, I. G.; Loza-Alvarez, P.; Gualda, E. J.; Artigas, D. Ultrashort pulse characterisation with SHG collinear-FROG. *Opt. Express* **2004**, *12*, 1169-1178. DOI: 10.1364/OPEX.12.001169.

(2) Stibenz, G.; Steinmeyer, G. Interferometric frequency-resolved optical gating. *Opt. Express* **2005**, *13*, 2617-2626. DOI: 10.1364/OPEX.13.002617.
